# Automated Analysis of Soft Matter Interfaces, Interactions, and Self-Assembly with PySoftK

**DOI:** 10.1021/acs.jcim.4c01849

**Published:** 2025-02-10

**Authors:** Raquel López-Ríos de Castro, Alejandro Santana-Bonilla, Robert M. Ziolek, Christian D. Lorenz

**Affiliations:** † Department of Chemistry, King’s College London, London SE1 1DB, United Kingdom; ‡ Biological Physics and Soft Matter Group, Department of Physics, King’s College London, London WC2R 2LS, United Kingdom; ¶ In Silico Toxicology and Structural Bioinformatics, Institute of Physiology, Charité-Universitätsmedizin Berlin, 10117 Berlin, Germany; § Department of Physics, King’s College London, London WC2R 2LS, United Kingdom; ∥ Department of Engineering, King’s College London, London WC2R 2LS, United Kingdom

## Abstract

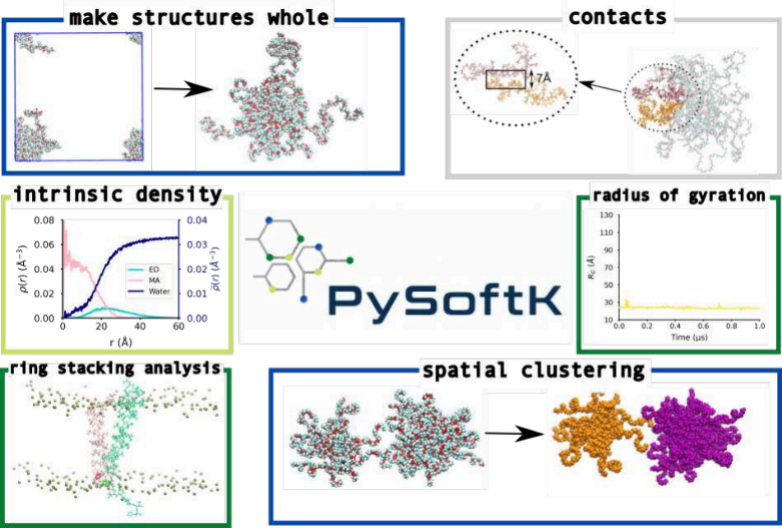

Molecular dynamics simulations have become essential tools in the study of soft matter and biological macromolecules. The large amount of high-dimensional data associated with such simulations does not straightforwardly elucidate the atomistic mechanisms that underlie complex materials and molecular processes. Analysis of these simulations is complicated: the dynamics intrinsic to soft matter simulations necessitates careful application of specific, and often complex, algorithms to extract meaningful molecular scale understanding. There is an ongoing need for high-quality automated computational workflows to facilitate this analysis in a reproducible manner with minimal user input. In this work, we introduce a series of molecular simulation analysis tools for investigating interfaces, molecular interactions (including ring–ring stacking), and self-assembly. In addition, we include a number of auxiliary tools, including a useful function to unwrap molecular structures that are greater than half the length of their corresponding simulation box. These tools are contained in the PySoftK software package, making the application of these algorithms straightforward for the user. These new simulation analysis tools within PySoftK will support high-quality, reproducible analysis of soft matter and biomolecular simulations to bring about new predictive understanding in nano- and biotechnology.

## Introduction

Soft matter spans materials science applications in cosmetics,^1−3^ pharmaceuticals,^4−7^ and water decontamination^8^ among many others. Advances in synthetic chemistry and formulation science have led to the development of a plethora of soft matter architectures, while ever-increasing computational power and simulation techniques have opened up opportunities for their study *in silico*. An understanding of the interplay of molecular structure, conformational dynamics and intermolecular interactions of the constituent molecules is required to build up generalizable structure–property relationships to support the rational design of new functional soft matter materials.

Molecular dynamics (MD) simulations provide the framework to investigate the molecular structure, dynamics, and interactions at a level of detail that cannot be resolved experimentally, such as in the field of soft matter self-assembly.^9−16^ These simulations generate a vast amount of high-dimensional data, but it is typically challenging to extract meaningful understanding of the underlying mechanisms at play. Interpreting the molecular mechanisms present in MD simulations often necessitates the development of bespoke computational tools, and as a result, it is often not possible to reproduce quantitative results.^17^

The computational soft matter community has invested significant effort in simplifying input creation for soft matter simulations, as exemplified by tools such as PySoftK,^18^ Polymer Structure Predictor,^19^ Radonpy^20^ and MoSDeF.^21^ However, a comprehensive package for analyzing soft matter material properties has not yet been reported. To address this issue, PySoftK (version 1.0) now includes a toolkit designed for analysis of soft matter simulations, providing a unified computational framework in which modeling and analysis can be streamlined under modern software development standards. These features mitigate important data provenance and reproducibility concerns. In line with the design commitment of PySoftK to minimize user inputs and provide highly efficient code, PySoftK v1.0 enables the analysis of large-scale soft matter systems.

This work introduces new computational analysis algorithms with illustrative case studies. The tools that have been included in the most recent release of PySoftK allow users to quantify the self-assembly of soft and biological molecules. For the self-assembly that results in sphere-like nanoparticles, there are tools that allow the user to accurately describe the interface of the nanoparticles, which in turns allows users to accurately understand the location of molecules within the nanoparticle and those near the nanoparticle’s interface. Additionally, we included functions that will allow the user to determine the size and shape of the nanoparticles. Also there is a tool that accurately takes into account periodic boundary conditions when modeling a collection of self-assembled molecules, where other tools are unable to do so. Finally, PySoftK will measure interactions between the self-assembled molecules and their solvent environment, and also it has a tool that can measure the specific interactions formed between ring containing molecules. These tools allow the user to identify the interactions which play a key role in the self-assembly and stabilizing of the self-assembled structure that is formed. These tools have not previously been shared with the community through a freely available and well-tested software package. PySoftK v1.0 provides an automated approach to investigate interfaces in soft matter systems, interactions that govern structure within soft matter, and soft matter self-assembly. PySoftK v1.0 supports users to investigate soft matter systems forming any nanoparticle or interface since the implemented algorithms are entirely chemically agnostic. With this new release, PySoftK version 1.0 further supports the acceleration of computer-aided materials development.

## Results and Discussion

The PySoftK package and associated tutorial notebooks are freely available on Github (https://github.com/alejandrosantanabonilla/pysoftk). The repository also contains short trajectories to run the tutorials and also to generate the figures presented in this manuscript. The majority of the molecular simulations analyzed with PySoftK within this work were originally published elsewhere. In each case, the original article is cited in the associated figure caption for clarity. Since PySoftK utilizes MDAnalysis^22^ to load topology and trajectory information, its functionality can be applied to the wide range of file formats supported by MDAnalysis.

### Analyzing Interfaces

In order to accurately understand the interfacial properties of nanoparticles, one must first be able to accurately describe the geometry of the interface of interest. Then the interfacial properties can be measured as well as the internal distribution of the components within a nanoparticle, which provides precise information regarding its structure.

#### Spherical Density

For nanoparticles that are approximately spherical, the interface of the nanoparticle can be described by identifying the radius of the nanoparticle from the particle’s center of mass. The density of its various components is calculated with reference to the aggregate’s center of mass. There are numerous MD studies that measure the density of components of polymer micelles using this approach,^12,23,24^ but there are limited open-source tools to reliably carry out such analysis. The PySoftK spherical_density tool, which is described in detail in the Electronic Supporting Information (ESI) section on spherical_density, allows users to easily calculate the spherical density over a trajectory, including structures undergoing molecular exchange during their simulation.

#### Intrinsic Density

**Figure 1:**
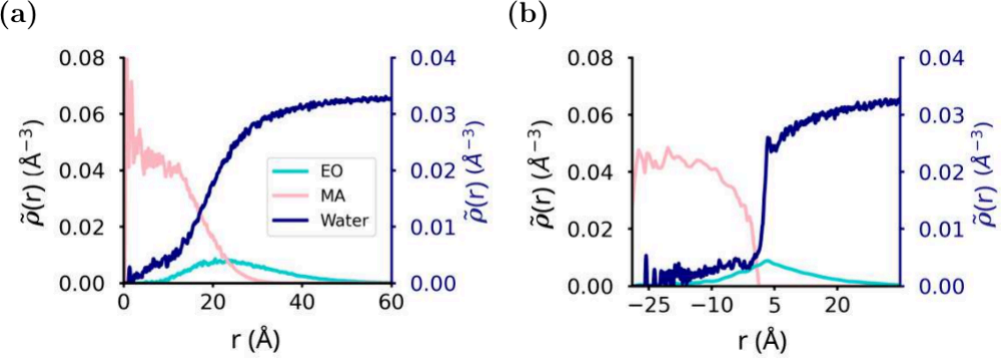
Comparing spherical and intrinsic density calculations. Density calculation of a spherical micelle formed by poly(ethylene oxide) and poly(methyl acrylate) block (PEO–PMA) polymers using (a) spherical_density and (b) intrinsic_density. Polymer trajectories of diblock PEO–PMA polymer were originally published elsewhere.^16^

If a nanoparticle possesses either significant asphericity or a rough interface, then defining a spherical interface will lead to misleading characterization. As a result, intrinsic interface approaches have been developed to identify the location of the uneven interfaces of nanoparticles.^25^ The intrinsic core–shell interface (ICSI) algorithm is one such example, which was originally developed to study core–shell micellar structures.^12,26^ This approach categorizes constituent molecules in a micelle into core and shell regions, automatically identifying the boundary between them (the core–shell interface). The intrinsic density of the nanoparticle is computed as the ratio of the relative positions of atoms with respect to the ICSI, normalized by the average volume of the shell in which a given atom is found. The normalization factor cannot be calculated analytically; therefore, we use Monte Carlo integration to calculate it. PySoftK’s intrinsic_density class harnesses the ICSI method to perform intrinsic density calculations. The implementation importantly enables seamless processing of the unwrapped coordinates provided by make_micelle_whole and is suitable for use on structures undergoing molecular exchange. The use of this function closely follows that of the spherical density function. Additionally, there is an intrinsic_density_water class to compute the intrinsic density of water, which requires different treatment. The intrinsic_density class outputs a NumPy array containing the intrinsic density with respect to the distance to the core–shell interface, where a distance *r* = 0 reflects the position of the interface. It also outputs another NumPy array with the values of the bins used in the density calculation. Figure 1 shows both spherical and intrinsic density calculations of the same diblock polymer nanoparticle. The spherical density tool outputs the density as a function of distance from the center of mass of the aggregate (Figure 1a), while the intrinsic density outputs the density as a function of the distance from the core–shell interface (Figure 1b). Negative distance values in the intrinsic density represent the core region. Both density plots share some conserved features, since the nanoparticle has a well-defined core–shell interface. From the spherical density plot, we can infer that the core of the nanoparticle is predominately formed of MA with an EO corona. We can also suggest that some water penetrates the core of the nanoparticle too. The intrinsic density plots confirm these deductions categorically, with the core formed mostly of MA with some EO blocks found throughout the nanoparticle core. Water clearly penetrates into the nanoparticle core (this can be measured and compared to the experimental results). The detailed interfacial structure of water is only apparent in Figure 1b, showing a small peak in the water intrinsic density plot at *r* ≈ 5 Å, indicative of a weakly hydrophobic interface.

### Molecular-Scale Interactions

This section describes tools to analyze different intermolecular interactions that play important roles in the self-assembly of a soft matter. We have developed novel methods for investigating ring stacking interactions, which are commonly found within aggregates of conjugated polymers, proteins, and other biopolymers. We also introduce a tool to automatically assess the solvation of different regions of the molecules.

#### Ring Stacking Analysis

Ring stacking interactions are a common driving force behind many collective phenomena ranging from DNA base pairing^27^ and protein–drug binding^28^ to through-space charge transfer in conjugated polymers.^29^

A class to identify ring–ring interactions has been developed for PySoftK v1.0. ProLIF can also calculate ring stacking interactions in protein–ligand systems.^30^ The algorithm implemented in PySoftK v1.0. has been specifically engineered for large soft matter systems, where ring stacking interactions across collections of molecules can be present. PySoftK’s ring stacking interaction algorithm consists of three stages. First, all atoms belonging to rings within the chosen molecules are automatically detected. Pairs of molecules within the system are then screened using a cutoff distance to define rings in close contact (distance between the center of geometry of two rings <10 Å). Finally, those rings in close contact are selected to have the necessary geometrical properties between the rings computed in the final stage (distance of any two atoms in the two rings <4 Å and an angular cutoff of 20° between the planes of each ring). Default values for the parameters were assigned with reference to our previous work.^31^ The algorithm is explained in more detail in the ESI (section RSA: Ring Stacking Analysis), and it is used to investigate an amorphous polymer system and the protein complex formed between TREM2 and DAP12^32^ as shown in Figure S16.

#### Automated Characterization of General Molecular Interactions

We have developed a code that allows a more general assessment of other types of interactions between molecules within the simulated system. One example is solvation analysis, which plays a crucial role in understanding the structure and dynamics of amphiphilic soft matter. This analysis allows us to quantify the solvation cells around molecules, and to predict hydrophobic interactions.^16,33^ PySoftK’s solvation class provides a straightforward method for quantifying solvation by determining the number of solvent molecules within the first solvation shell of the specified molecules. In doing so, PySoftK will identify the solvent molecules that are hydrogen-bonded to a particular part of a molecule, as well as any that might be attracted via other types of interactions (e.g., electrostatic, hydrophobic), and thus is a more general way of identifying the solvation of different parts of molecules. Quantifying intermolecular interactions is vital to understanding the mechanisms that drive molecular-scale phenomena. The contacts tool calculates the contacts between molecules by measuring the distance between selected atoms. If the intermolecular distance between two selected atoms is less than a user-defined cutoff, then it is considered a contact. More details regarding the implementation and application of the solvation and contact analysis tools within PySoftK are included in the ESI (solvation and contacts: Quantification of intermolecular interactions sections, respectively).

### Tracking Self-Assembly

The algorithms introduced in this section are useful for performing the analysis of soft matter aggregates, including self-assembly. The algorithms are entirely resolution and chemically agnostic.

#### Tracking Self-Assembly

**Figure 2:**
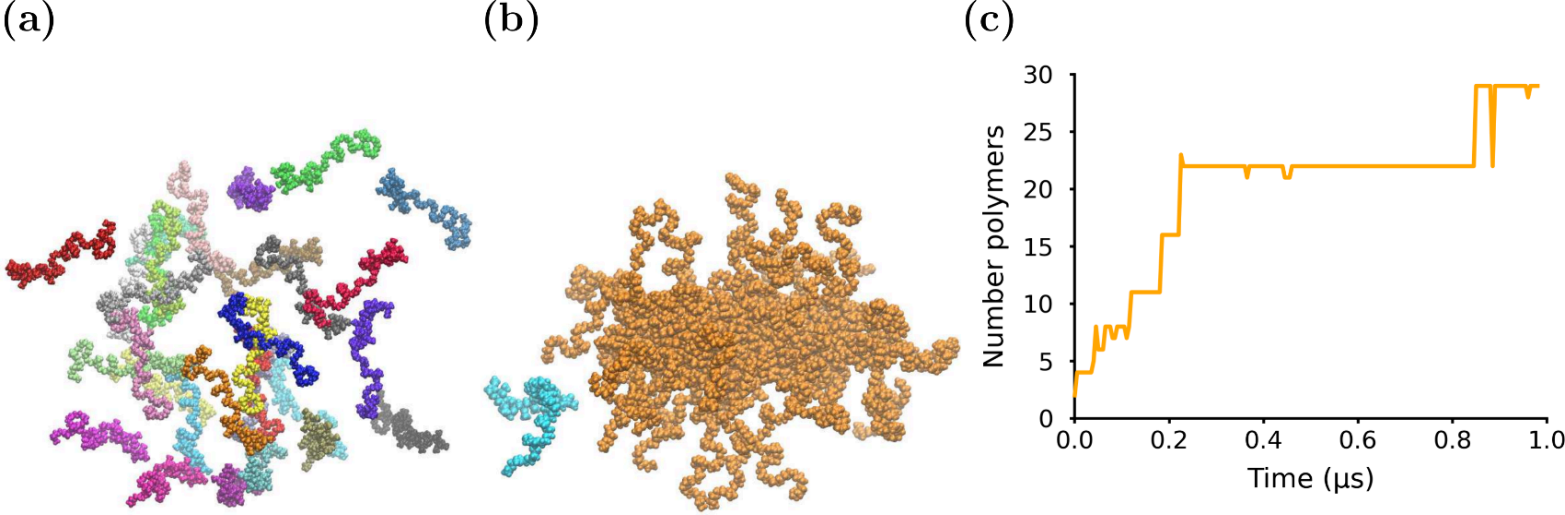
Analyzing the self-assembly of soft matter. (a) Snapshot from the simulation of randomly distributed diblock PEO–PMA polymer molecules in solution (note: water is not shown so that the polymers can be seen clearly). (b) Snapshot of the simulated system after the polymers have formed one large (orange) micelle and another small (cyan) micelle. (c) Plot of the number of polymer molecules in the largest aggregate as a function of time in the simulation. Polymer trajectories of diblock PEO–PMA polymer were originally published elsewhere.^16^

The Spatial Clustering Protocol (SCP) algorithm provides a fast way to label molecules based on the cluster or aggregate in which they reside during a self-assembly process. We make use of simple graph theory to represent an aggregate of molecules as a graph, where each molecule is a node, and if any two molecules are within the defined distance cutoff, an edge is added between these two nodes in the graph. In this representation, clusters are rapidly identified as connected subgraphs. This makes this analysis suitably fast such that the dynamical self-assembly process can be quickly rationalized over an entire trajectory. The algorithm returns a Pandas dataframe which contains the molecule residue IDs for each cluster and the cluster size for each time step. More details about the application of graph theory to investigating self-assembly can be found in the ESI (section SCP: Spatial clustering of polymers). Figure 2 demonstrates PySoftK’s SCP algorithm applied to self-assembling diblock polymers, which can be used to track the exchange of unimers between different aggregates during the simulation.

#### Unwrapping Large Aggregates across the PBC

Upon self-assembly, the resultant nanoparticle may span more than half the length of the simulation box in one or more dimensions. In order to accurately analyze the nanoparticle and its environment, it is necessary to accurately represent the location of all of the molecules that make up the nanoparticle, while accounting for periodic boundary conditions. Various tools have been implemented elsewhere that can accurately reconstruct the position of molecules across the periodic boundary conditions (PBCs), but importantly, they fail to do so when molecular structures or molecules span a distance of more than half of the simulation box size in a given dimension.

**Figure 3:**
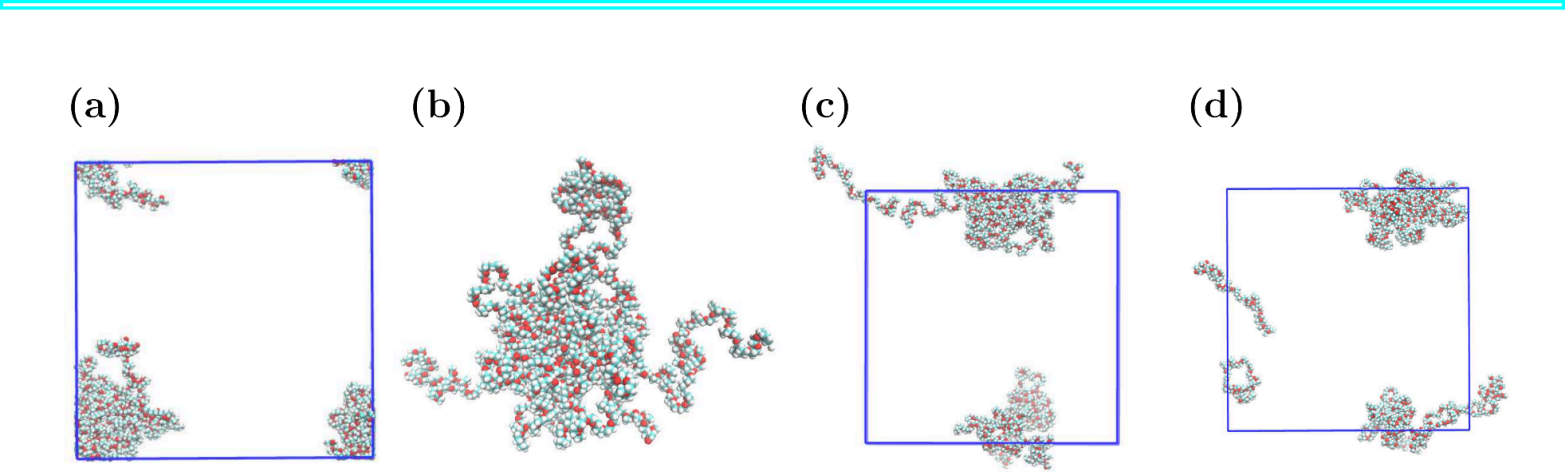
Unwrapping a polymer nanoparticle that is bigger than half the box length using PySoftK. (a) Polymer nanoparticle shown spanning the PBC, which is unwrapped using (b) PySoftK, (c) MDAnalysis, and (d) GROMACS 2023 (gmx trjconv -pbc mol). The polymer simulation of diblock PEO–PMA was originally published elsewhere.^16^

The make_micelle_whole tool in PySoftK can successfully reposition the molecules within a self-assembled aggregate if the aggregate is larger than half of the length of the simulation box in one or more dimensions. Figure 3a shows a polymer micelle that spans the simulation box in at least two dimensions. Figure 3b demonstrates the utility of make_micelle_whole, which successfully reconstructs the micelle shown in Figure 3a. Figure 3c,d demonstrates that the algorithms reported in MDAnalysis v2.5 and GROMACS 2023, respectively, are not able to reconstruct the micelle using the same input files as used with PySoftK’s make_micelle_whole function. The MDAnalysis unwrapping procedure is described in a PySoftK tutorial notebook (https://github.com/alejandrosantanabonilla/pysoftk/blob/main/pol_analysis_tutorials/example_mdanalysis_vs_micelle_whole.ipynb), which also includes a more detailed comparison of MDAnalysis and PySoftK unwrapping results. Also more details about the unwrapping procedure can be found in the ESI section make_micelle_whole. Our algorithm provides reliable and robust reconstruction of large molecular assemblies across periodic boundaries.

**Figure 4:**
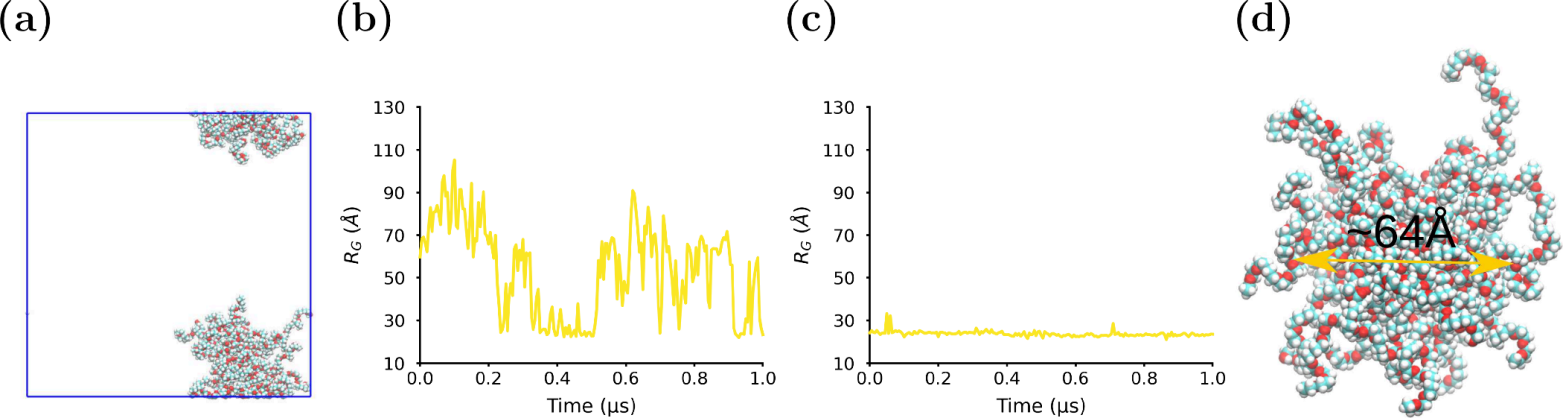
Effect of incorrect unwrapping on the calculation of radius of gyration. Result using MDAnalysis radius_of_gyration() on (a) the polymer nanoparticle spanning the PBC following unwrapping with (b) MDAnalysis and (c) PySoftK. (d) Snapshot of the reconstructed micelle with its diameter indicated (not to scale) for reference. Polymer trajectories of diblock PEO–PMA polymer were originally published elsewhere.^16^

As an example, a simple radius of gyration calculation can introduce analysis artifacts when computed on incorrectly unwrapped coordinates. Figure 4 highlights the difference in radius of gyration calculated using polymer nanoparticle coordinates unwrapped using MDAnalysis and PySoftK.

#### Auxiliary Functions

For convenience, we also included specific functions to calculate the radius of gyration and eccentricity of self-assembled structures. These are described in the ESI (sections rgyr: radius of gyration and ecc: Eccentricity calculation).

## Conclusion

PySoftK v1.0 adds a complete standalone module for the analysis of soft matter systems. This work provides a set of interconnected tools that are useful for determining the physical properties of soft matter self-assembled aggregates as well as the molecular-scale interactions that underlie such emergent behavior. One of the key features of PySoftK v1.0 is that it properly accounts for periodic boundary conditions when determining the positions of atoms within the molecules that make up a soft matter aggregate if the aggregate is larger than half the size of the simulation box in one or multiple dimensions (as is typical in soft matter self-assembly simulations). Other software tools are not designed to account for molecular assemblies of this large size. A thorough suite of tests has been created to cover the code to ensure its correct functionality. Furthermore, PySoftK version 1.0 is designed to provide maximum flexibility to the user. Most functions output the data per outputted configuration of the trajectory so that the user can decide how to represent or further process the data. Although the initial version of PySoftK has a particular focus on polymers, the analysis module has been created such that it is fully chemically agnostic. PySoftK v1.0 is an open-source platform that allows users to analyze complex structures, dynamics, and interactions in their simulations with minimal user input. PySoftK v1.0 contributes to the standardization of molecular-scale simulation analysis, which will promote accurate comparisons across different simulations to support the rational *in silico* design of new soft materials.

## Data Availability

PySoftK v1.0 is freely available at: https://github.com/alejandrosantanabonilla/pysoftk.

## Supporting Information Available

Description of application of graph theory to molecular simulations. Description of PySoftK’s algorithm that employs graph theory to analyze self-assembly of soft matter. Description of PySoftK development. Detailed description and examples of each analysis tool within PySoftK. (PDF)

## References

[ref1] Mitura S.; Sionkowska A.; Jaiswal A. Biopolymers for hydrogels in cosmetics: review. J. Mater. Sci.: Mater. Med. 2020, 31, 50 10.1007/s10856-020-06390-w. 32451785 PMC7248025

[ref2] Ahmadi D.; Ledder R.; Mahmoudi N.; Li P.; Tellam J.; Robinson D.; Heenan R. K.; Smith P.; Lorenz C. D.; Barlow D. J.; Lawrence M. J. Supramolecular architecture of a multi-component biomimetic lipid barrier formulation. J. Colloid Interface Sci. 2021, 587, 597–612. 10.1016/j.jcis.2020.11.017. 33239213

[ref3] Gupta S.; Sharma S.; Kumar Nadda A.; Saad Bala Husain M.; Gupta A. Biopolymers from waste biomass and its applications in the cosmetic industry: A review. Mater. Today: Proc. 2022, 68, 873–879. 10.1016/j.matpr.2022.06.422.

[ref4] Kohli A. G.; Kierstead P. H.; Venditto V. J.; Walsh C. L.; Szoka F. C. Designer lipids for drug delivery: From heads to tails. J. Controlled Release 2014, 190, 274–287. 10.1016/j.jconrel.2014.04.047. PMC4142081 24816069

[ref5] Chen C. H.; Liu Y.-H.; Eskandari A.; Ghimire J.; Lin L. C.-W.; Fang Z.-S.; Wimley W. C.; Ulmschneider J. P.; Suntharalingam K.; Hu C.-M. J.; Ulmschneider M. B. Integrated Design of a Membrane-Lytic Peptide-Based Intravenous Nanotherapeutic Suppresses Triple-Negative Breast Cancer. Advanced Science 2022, 9, 2105506 10.1002/advs.202105506. 35246961 PMC9069370

[ref6] Ishkhanyan H.; Rhys N. H.; Barlow D. J.; Lawrence M. J.; Lorenz C. D. Impact of drug aggregation on the structural and dynamic properties of Triton X-100 micelles. Nanoscale 2022, 14, 5392 10.1039/D1NR07936K. 35319029

[ref7] Saaka Y.; Allen D.; Terry A. E.; Lorenz C. D.; Barlow D. J.; Lawrence M. J. Characterisation of the apparent aqueous solubility enhancement of testosterone analogues in micelles of dodecyl-chained surfactants with different headgroups. J. Mol. Liq. 2023, 385, 122376 10.1016/j.molliq.2023.122376.

[ref8] Shah A.; Shahzad S.; Munir A.; Nadagouda M. N.; Khan G. S.; Shams D. F.; Dionysiou D. D.; Rana U. A. Micelles as Soil and Water Decontamination Agents. Chem. Rev. 2016, 116, 6042–6074. 10.1021/acs.chemrev.6b00132. 27136750

[ref9] Srinivas G.; Discher D. E.; Klein M. L. Self-assembly and properties of diblock copolymers by coarse-grain molecular dynamics. Nature materials 2004, 3, 638–644. 10.1038/nmat1185. 15300242

[ref10] Jorge M. Molecular Dynamics Simulation of Self-Assembly of *n*-Decyltrimethylammonium Bromide Micelles. Langmuir 2008, 24, 5714–5725. 10.1021/la800291p. 18454560

[ref11] Wang H.; Zhang H.; Liu C.; Yuan S. Coarse-grained molecular dynamics simulation of self-assembly of polyacrylamide and sodium dodecylsulfate in aqueous solution. J. Colloid Interface Sci. 2012, 386, 205–211. 10.1016/j.jcis.2012.07.026. 22901375

[ref12] Ziolek R. M.; Omar J.; Hu W.; Porcar L.; Gonzalez-Gaitano G.; Dreiss C. A.; Lorenz C. D. Understanding the pH-directed self-assembly of a four-arm block copolymer. Macromolecules 2020, 53, 11065–11076. 10.1021/acs.macromol.0c01694.

[ref13] Pink D. L.; Foglia F.; Barlow D. J.; Lawrence M. J.; Lorenz C. D. The Impact of Lipid Digestion on the Dynamic and Structural Properties of Micelles. Small 2021, 17, 2004761 10.1002/smll.202004761. 33470509 PMC11475325

[ref14] Pink D. L.; Loruthai O.; Ziolek R. M.; Terry A. E.; Barlow D. J.; Lawrence M. J.; Lorenz C. D. Interplay of lipid and surfactant: Impact on nanoparticle structure. J. Colloid Interface Sci. 2021, 597, 278–288. 10.1016/j.jcis.2021.03.136. 33872884

[ref15] Bhendale M.; Singh J. K. Molecular Insights on Morphology, Composition, and Stability of Mixed Micelles Formed by Ionic Surfactant and Nonionic Block Copolymer in Water Using Coarse-Grained Molecular Dynamics Simulations. Langmuir 2023, 39, 5031–5040. 10.1021/acs.langmuir.3c00045. 36992607

[ref16] Lopez-Rios de Castro R.; Ziolek R. M.; Lorenz C. D. Topology-Controlled Self-Assembly of Amphiphilic Block Copolymers. Nanoscale 2023, 15, 15230 10.1039/D3NR01204B. 37671739 PMC10540979

[ref17] Gartner T. E. III; Jayaraman A. Modeling and simulations of polymers: a roadmap. Macromolecules 2019, 52, 755–786. 10.1021/acs.macromol.8b01836.

[ref18] Santana-Bonilla A.; Lopez-Rios de Castro R.; Sun P.; Ziolek R. M.; Lorenz C. D. Modular Software for Generating and Modeling Diverse Polymer Databases. J. Chem. Inf. Model. 2023, 63, 3761 10.1021/acs.jcim.3c00081. 37288782 PMC10302471

[ref19] Sahu H.; Shen K.-H.; Montoya J. H.; Tran H.; Ramprasad R. Polymer structure predictor (psp): a python toolkit for predicting atomic-level structural models for a range of polymer geometries. J. Chem. Theory Comput. 2022, 18, 2737–2748. 10.1021/acs.jctc.2c00022. 35244397

[ref20] Hayashi Y.; Shiomi J.; Morikawa J.; Yoshida R. RadonPy: automated physical property calculation using all-atom classical molecular dynamics simulations for polymer informatics. npj Computational Materials 2022, 8, 222 10.1038/s41524-022-00906-4.

[ref21] Summers A. Z.; Gilmer J. B.; Iacovella C. R.; Cummings P. T.; McCabe C. MoSDeF, a Python Framework Enabling Large-Scale Computational Screening of Soft Matter: Application to Chemistry-Property Relationships in Lubricating Monolayer Films. J. Chem. Theory Comput. 2020, 16, 1779–1793. 10.1021/acs.jctc.9b01183. 32004433

[ref22] Michaud-Agrawal N.; Denning E. J.; Woolf T. B.; Beckstein O. MDAnalysis: a toolkit for the analysis of molecular dynamics simulations. Journal of computational chemistry 2011, 32, 2319–2327. 10.1002/jcc.21787. 21500218 PMC3144279

[ref23] Kovacevic M.; Balaz I.; Marson D.; Laurini E.; Jovic B. Mixed-monolayer functionalized gold nanoparticles for cancer treatment: Atomistic molecular dynamics simulations study. Biosystems 2021, 202, 104354 10.1016/j.biosystems.2021.104354. 33444701

[ref24] Wilkosz N.; Łazarski G.; Kovacik L.; Gargas P.; Nowakowska M.; Jamroz D.; Kepczynski M. Molecular insight into drug-loading capacity of PEG–PLGA nanoparticles for itraconazole. J. Phys. Chem. B 2018, 122, 7080–7090. 10.1021/acs.jpcb.8b03742. 29927603

[ref25] Sega M.; Kantorovich S. S.; Jedlovszky P.; Jorge M. The generalized identification of truly interfacial molecules (ITIM) algorithm for nonplanar interfaces. J. Chem. Phys. 2013, 138, 044110 10.1063/1.4776196. 23387571

[ref26] Ziolek R. M.; Smith P.; Pink D. L.; Dreiss C. A.; Lorenz C. D. Unsupervised learning unravels the structure of four-arm and linear block copolymer micelles. Macromolecules 2021, 54, 3755–3768. 10.1021/acs.macromol.0c02523.

[ref27] Kool E. T. Hydrogen bonding, base stacking, and steric effects in DNA replication. Annual review of biophysics and biomolecular structure 2001, 30, 1–22. 10.1146/annurev.biophys.30.1.1. 11340050

[ref28] Liu Y.; Liu B.-Y.; Hao P.; Li X.; Li Y.-X.; Wang J.-F. *π–π* Stacking mediated drug–drug interactions in human CYP2E1. Proteins: Struct., Funct., Bioinf. 2013, 81, 945–954. 10.1002/prot.24260. 23349037

[ref29] Schwartz B. J. Conjugated polymers as molecular materials: How chain conformation and film morphology influence energy transfer and interchain interactions. Annu. Rev. Phys. Chem. 2003, 54, 141–172. 10.1146/annurev.physchem.54.011002.103811. 12524429

[ref30] Bouysset C.; Fiorucci S. ProLIF: a library to encode molecular interactions as fingerprints. Journal of Cheminformatics 2021, 13, 72 10.1186/s13321-021-00548-6. 34563256 PMC8466659

[ref31] Ziolek R. M.; Santana-Bonilla A.; Lopez-Rios de Castro R.; Kuhn R.; Green M.; Lorenz C. D. Conformational heterogeneity and interchain percolation revealed in an amorphous conjugated polymer. ACS Nano 2022, 16, 14432–14442. 10.1021/acsnano.2c04794. 36103148 PMC9527807

[ref32] Zhong Z.; Ulmschneider M. B.; Lorenz C. D. Unraveling the Molecular Dance: Insights into TREM2/DAP12 Complex Formation in Alzheimer’s Disease through Molecular Dynamics Simulations. ACS Omega 2024, 9, 28715–28725. 10.1021/acsomega.4c03060. 38973875 PMC11223195

[ref33] Jafari M.; Doustdar F.; Mehrnejad F. Molecular self-assembly strategy for encapsulation of an amphipathic α-helical antimicrobial peptide into the different polymeric and copolymeric nanoparticles. J. Chem. Inf. Model. 2019, 59, 550–563. 10.1021/acs.jcim.8b00641. 30475620

[ref34] King’s College London King’s Computational Research, Engineering and Technology Environment (CREATE) 2022; 10.18742/rnvf-m076.

