# Supplementary material for: Automated Analysis of Soft Matter Interfaces, Interactions, and Self-Assembly with PySoftK
Source: J Chem Inf Model. 2025 Feb 10;65(4):1679–84. doi: 10.1021/acs.jcim.4c01849 (PMC11863363; doi:10.1021/acs.jcim.4c01849)
Supplement: Supplementary file 1 — ci4c01849_si_001.pdf [file ci4c01849_si_001.pdf]

Supplementary information for "Automated Analysis of Soft Matter Interfaces, Interactions and Self-Assembly with PySoftK"

Raquel López-Ríos de Castro,[†,‡,¶] Alejandro Santana-Bonilla,[*,§] Robert M. Ziolek,[‡,‖] and Christian D. Lorenz[*,‡,‖]

†*Department of Chemistry, King's College London, London, SE1 1DB, United Kingdom*

‡*Biological Physics and Soft Matter Group, Department of Physics, King's College London, London, WC2R 2LS, United Kingdom*

¶*In Silico Toxicology and Structural Bioinformatics, Institute of Physiology, Charité-Universitätsmedizin Berlin, 10117, Berlin, Germany*

§*Department of Physics, King's College London, London, WC2R 2LS, United Kingdom*

‖*Department of Engineering, King's College London, London, WC2R 2LS, United Kingdom*

E-mail: alejandro.santana_bonilla@kcl.ac.uk; chris.lorenz@kcl.ac.uk

Applications of Graph Theory to Molecular Simulations

MD simulations generate large amounts of data that need to be analysed in order to understand the biophysics of the system. There are many different techniques and software that extract the time evolution of specific structural properties, like hydrogen-bond distances, radial distribution functions of atoms or root mean square displacement of global structures (RMSD). However, there are not that many computational analysis methods that provide a direct knowledge of the changes in the global 3-d structures over time.[1] For this purpose,

graph theory can be of use, since this is a mathematical framework that analyzes relationships between entities, often represented as nodes, and their interactions, represented as edges, which can be updated across time steps. Furthermore, graph theory is computationally very efficient, so it can be used for large system with small computational cost.[1]

As its core, a graph $G(V, E)$ is made up of vertices V (also known as nodes), that are connected by edges E. These nodes can represent different entities, either single atoms or the center of mass of a molecule. The edges are the relationships, interactions or connections that exist between these nodes, for example covalent bonds, Hydrogen bonds or a simple distance criteria. Therefore, one can use graph theory to track global structural changes, since the high dimensionality of the simulation trajectory can be reduced by examining the network connectivity between the nodes and edges changes throughout the simulation. The information to create the graph is obtained from the adjacency matrix A. The adjacency matrix is a symmetric matrix that summarizes the network connectivity information of a graph. Figure S1 shows an example of how the connectivity of a graph can be obtained from the adjacency matrix.

(a)

$$A = \begin{bmatrix} 0 & 1 & 1 & 0 \\ 1 & 0 & 0 & 1 \\ 1 & 0 & 0 & 0 \\ 0 & 1 & 0 & 0 \end{bmatrix}$$

(b)

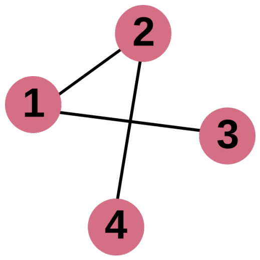

Figure S1: **Conversion from adjacency matrix A to Graph $G(V, E)$.** (a) Adjacency matrix A and (b) its corresponding graph representation.

In Figure S1 (a) each element of the matrix is an element of the graph. If the element A_{ij} of the matrix is 1, it means that nodes i and j are connected with an edge, while if it is 0 it means they are not connected. For example, the first row of the matrix in Figure S1 (a) has 1s in the second and third element, meaning that element 1 is connected to element 2 and 3, as depicted in Figure S1 (b). This type of matrix, where the edges are just 0s and 1s,

so that the edges have no associated weights to them, are called unweighted graphs. These are the type of graphs that will be used in PySoftK v1.0. The use of graph theory to analyse molecular simulations is not new, it has been used to track hydrogen bonding,[2] studying the solvation of lipid headgroups[3] or to study molecular isomorphism[1] among many other physical and chemical properties. However, there is currently no openly available software that uses graph theory to study the self-assembly of soft NP. All graph-based analysis has been performed with the Python library Networkx.[4]

Graph theoretical cluster algorithm

Studying the structure of the self-assembly of micelles is not trivial. Polymers do not tend to form perfect NP structures, where all polymers are clearly packed in the same way during the whole simulation. Actually, it is quite common for polymers to not only form one micelle, but to form various clusters within the simulation box and for these clusters to fluctuate over time. This makes the analysis of the simulation more difficult, since always selecting the largest or most stable micelle in each time step separately is not trivial, and there is currently no openly available software to do this. Thus, a novel graph theory-based clustering algorithm is developed, and has been introduced into the software package PySoftK so that it is openly available to analyse any simulations where there are aggregates formed by lipids, polymers, proteins, etc.

This method uses an unweighted undirected graph $G(V, E)$, where V are individual polymers and E are the interactions between them. These interactions are defined as a distance criteria, in such a way that if the distance between two molecules i and j, is less than the cut off distance (that is defined by the user), they are connected and form part of the same graph. The combination of connected subgraphs of G form the specific cluster at a given time. The nodes are also defined by the user. The user can define as many nodes to represent a molecule as they wish. For example, in the case of an amphiphilic molecule, each molecule

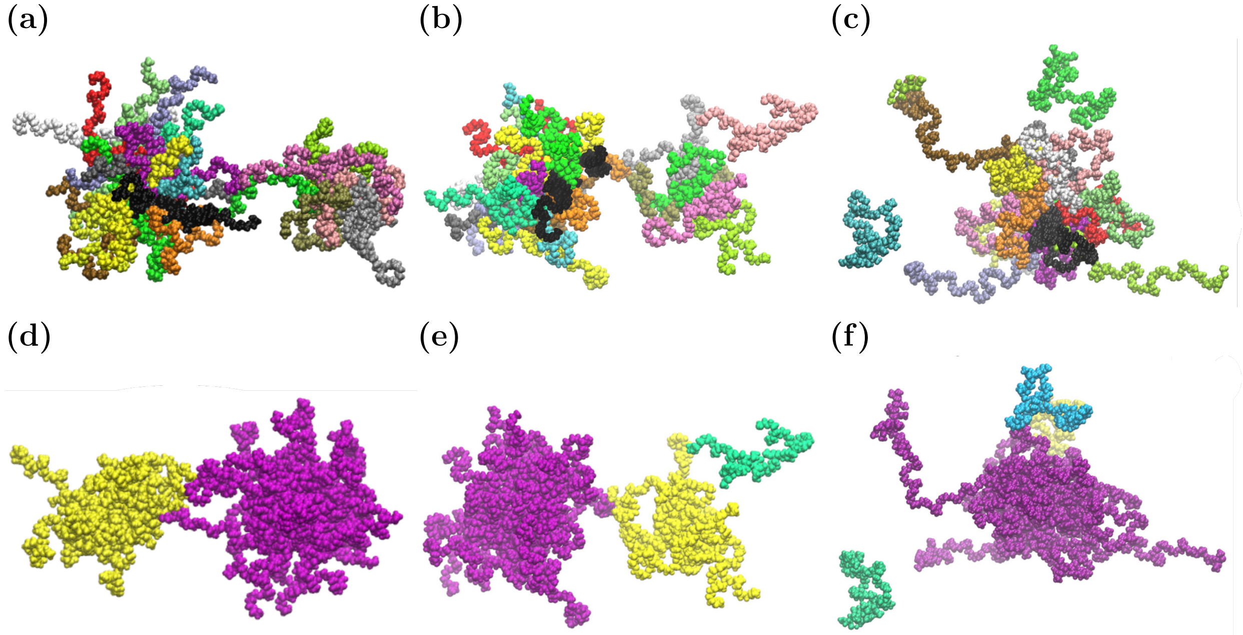

Figure S2: **Graph theoretic clustering algorithm applied on different polymer micelles.** (a) - (c) Plots of non-clustered micelles, where each color represents a different polymer. (d) - (f) Result of applying the clustering algorithm to each systems respectively. Polymers belonging to the same micelle have the same color. From here it is clear that the aggregates within the same system are distinctly separated. Simulation trajectory of self-assembling diblock PEO-PMA polymers taken from López-Ríos et al.[5]

can be defined with two different atoms, one in the hydrophobic part and the other in the hydrophilic part. Then, an edge is defined as the distance between the molecule i and molecule j using as reference point any of the user-selected two atoms of molecules i and any of the two atoms of molecule j and required to be less than the cutoff. Examples of the applications of this code can be seen in Figure S2, here (a) - (c) are snapshots of polymer simulations, where they do not form a clear defined single micelle. The results of the application of this algorithm to them are shown in (d) - (f), from here it is clear that the algorithm successfully identifies the clusters and groups them into the different aggregates and micelles present in the simulation, which facilitates the analysis of each micelle individually.

Software development

PySoftK v1.0 can be easily installed using the `pip` command strategy: `pip install pysoftk`. PySoftK v1.0 has been thoroughly tested and is compatible with Linux and macOS operating systems. It requires Python 3.7 or a higher version. Furthermore, PySoftK utilizes three python libraries: MDAnalysis v2.5,[6] pySoftWhere and Networkx.[4] In order to enhance computational efficiency of this module, parallel strategies have been implemented. These strategies utilize the `concurrent.futures` library or `MDAnalysis.lib.distances` function for parallel distance calculations.

PySoftK v1.0 has been built using modern code development practices. To ensure the preservation of functional software, Continuous Integration (CI) strategies have been implemented in PySoftK v1.0. Firstly, a peer-reviewed process through constant code committing has been carried out ensuring the detection of inconsistencies between versions of PySoftK. Secondly, a new set of tests has been developed and the new version of PySoftK has been used within each test ensuring its compatibility. Finally, code-coverage has been employed for ensuring the maximum compatibility between versions. The tests designed for PySoftK v1.0

rigorously check the functionality of all the new analysis tools. Each function is tested with various polymer types to ensure broad coverage of the analysis tools. Successful tests lead to continuous deployment, allowing for seamless updates of PySoftK. Additionally, jupyter notebook tutorials showing working examples (based on our tests) have been developed for all the analysis functions of PySoftK v1.0.

Software Overview

PySoftK v1.0 is a modular Python package that combines all of the functionality found within previous PySoftK versions[7] with an additional module named `pol_analysis`. This new module focuses on the analysis of soft matter simulations and allows the users to extract time dependent properties of these systems with minimal user input and high efficiency such that they can investigate the large systems which are now commonplace. The analysis tools of this module are divided into two categories, those that measure the properties of aggregates and those that measure molecular-scale interactions.

`SCP`: Spatial clustering of polymers.

The code in Figure S3 shows an example of how to use the `SCP` algorithm. From this code snippet, it is clear that to obtain the clustering of a simulation, apart from the atom names and the cutoff distance, all that is needed are the topology and trajectory of the simulation, the start and stop frames to define the time range for running the clustering algorithm, and the step parameter, which determines the number of frames to skip. Finally, `results_name` is the name of the output of the `SCP` function, which is a parquet file that contains a pandas data frame with the resids of the polymers grouped by the micelle they belong to per time step. Additionally, The code snippet in Figure S5 illustrates the output for the `SCP` clustering.

Figure S5 displays a pandas data frame containing three columns, the first one is the

```python
from pysoftk.pol_analysis.tools.utils_mda import MDA_input
from pysoftk.pol_analysis.tools.utils_tools import *
from pysoftk.pol_analysis.clustering import SCP

#Select trajectory
topology='topology.tpr'
trajectory='trajectory.xtc'

#Select output name
results_name='results_clustering'

#Select cluster cutoff distance
cluster_cutoff=12

#Select atom names
atom_names=['C02B', 'C01K', 'C02N']

#Select frames to run the clustering on
start=0
stop=10001
step=1

#Run clustering
clustering = SCP(topology, trajectory).spatial_clustering_run(
    start, stop, step, atom_names, cluster_cutoff, results_name)
```

Figure S3: Code snippet showing how to run the `SCP` function.

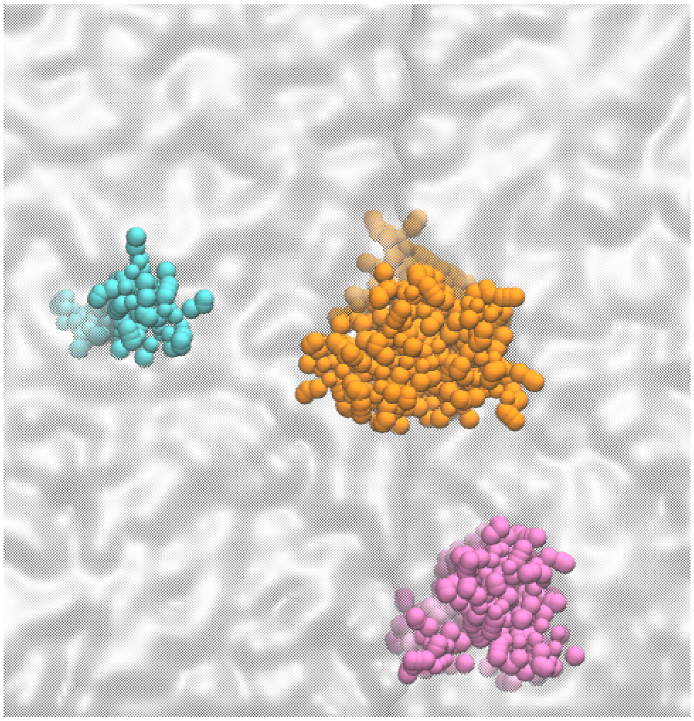

Figure S4: **SCP applied to a CG protein simulation to measure peptide aggregation.** Since the input needed to run this tool is not exclusive to polymers, it can be applied to any other type of system to measure molecular clustering. In this case, this is the result of the SCP being applied on CG transmembrane peptides inserted into a membrane. This is a top-view of the peptides-membrane system. Proteins colored in the same way belong to the same cluster. Blue cluster contains two peptides, the pink cluster has 6 peptides and the orange cluster has 8 peptides. The lipids membrane is colored in silver. The system was simulated using the MARTINI2 coarse-grained force field and comprised 16 APP transmembrane domain peptides in a lipid bilayer containing POPC lipids. Representation is not to scale.

time in ps, the second one is the resids of the polymers that belong to the same cluster. The polymers belonging to the same cluster are grouped by square brackets (a pythonic list). And the last column is the micelle size (number of polymers) of each of the clusters in the simulation. Note that the `SCP_tutorial` can be found on GitHub, with a step by step guide on how to use the SCP tool in two different case examples.

The choice of which atom to use in the identification of molecular contacts is specific to the molecule of interest. One can select as many atoms as necessary to accurately describe the self-assembly of the molecules. The SCP algorithm calculates the distances between all the selected atoms of the chosen molecules. If any of these distances is below the cutoff it will

```
time    micelle_resids                              micelle_size
0.0     [[1, 2, 3, 4, 5, 6, 7, 8, 9, 10]]           [10]
100.0   [[1, 2, 3, 4, 5, 6, 7], [8, 9], [10]]]      [7, 2, 1,]
```

Figure S5: Code snippet showing the output of the SCP function.

then add them to the same subgraph. It is important to note that choosing a large number of atoms will slow down this calculation. Figure S6 shows how different atom selection choices affect the output of the SCP clustering for an ABA triblock copolymer. Figure S6 (a) shows the system, two micelles formed by ABA triblock polymers (the A block is hydrophilic; the B block is hydrophobic). Figure S6 (b) displays the desired clustering output. This result is achieved by selecting the backbone C atom of the middle monomer of the hydrophobic block. Since the hydrophobic block tightly interacts with those of other polymers in the micelle, picking atoms within this domain is a reasonable selection. On the other hand, Figure S6 (c) shows the clustering performed for the same system but picking atoms at the end of the hydrophilic blocks. The hydrophilic atoms at the end of the polymer chains are not suitable choices for clustering and as such, the clusters obtained do not reflect the formation of two micelles.

Apart from selecting atoms, users must also specify a cutoff distance for clustering. This distance determines whether two molecules belong to the same cluster. The cutoff distance might be obtained from the radial distribution function (RDF) of the selected atoms (either the position of the RDF maximum or first minimum). The lack of a single clear choice for the cutoff distance is caused by the complex structures of self-assembled structures compared to, for examples, ions in solutions (i.e., the system is inherently not well-mixed). It is necessary to consider a range of cutoff distances, in our experience typically investigating a range of cutoffs between 8 Å and 13 Å is useful. Visual inspection of the resulting clusters determines the most appropriate cutoff distance straightforwardly in most cases. As well as soft matter, the SCP algorithm can be readily applied to biological systems, highlighting its broad applicability. The result of applying the SCP algorithm to a coarse-grained protein

(a) (b) (c)

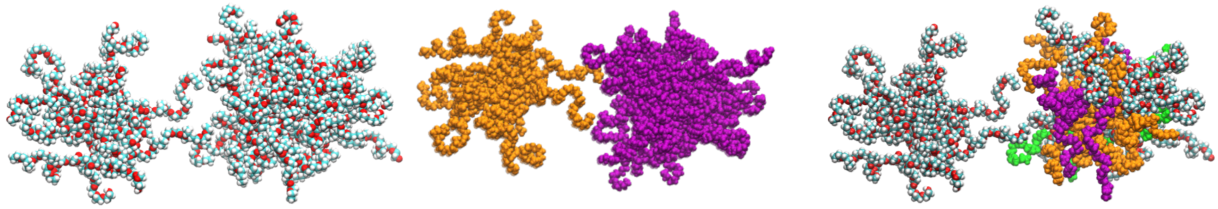

Figure S6: **Atom selection strongly influences the results of SCP clustering.** (a) Triblock hydrophilic terminated polymer system to cluster. (b) SCP algorithm applied on the system in (a) picking the middle hydrophobic monomer C atoms for the clustering. Polymers with the same color belong to the same cluster outputted by the algorithm. Clearly, the clustering here is done correctly. (c)SCP algorithm applied on the system in (a) picking the ending hydrophilic atoms. Polymers with the same color belong to the same cluster outputted by the algorithm. It is evident that the clustering is done badly when these atoms are used. The ending hydrophilic monomers do not play a key role in the intermolecular interactions with other polymers during the self-assembly of the micelle, so they are not good clustering candidates. Polymer trajectories of diblock PEO-PMA polymer from *López-Ríos et al.*[5]

simulation to measure the aggregation of the transmembrane domains of a protein within a lipid bilayer is shown Figure S4.

make_micelle_whole

The `make_micelle_whole` class is composed of three functions. The first one, `obtain_largest_micelle_resids`, can be used to extract the largest micelle polymer residues from the pandas dataframe obtained with `SCP`. This function was implemented because, in many cases, the structure of interest is the largest aggregate within the simulation. The code snippet in Figure S7 demonstrates how to use this tool. This Figure shows that the `obtain_largest_micelle_resids` function only requires the result from `SCP` as input. It then produces a list, with as many entries as time steps evaluated, containing the resids of the molecules that belong to the largest cluster at each time step. An illustrated and step-by-step guide on how to use all the functions in the `make_micelle_whole` class is provided in the jupyter notebook tutorial `micelle_whole_tutorial`. In this tutorial, it is shown how different polymer aggregates can be selected from the simulation, not just the largest micelle. In this sense, all the

```python
from pysoftk.pol_analysis.tools.utils_mda import MDA_input
from pysoftk.pol_analysis.tools.utils_tools import *
from pysoftk.pol_analysis.clustering import SCP
from pysoftk.pol_analysis.make_micelle_whole import micelle_whole

#Select trajectory
topology='topology.tpr'
trajectory='trajectory.xtc'

#Load clustering resids
resids_total='results_clustering.parquet'

#Run function
result = micelle_whole(topology, trajectory).
    obtain_largest_micelle_resids(resids_total)
```

Figure S7: Code snippet showing how to use `obtain_largest_micelle_resids`.

examples displayed are with the largest micelle for simplicity. The `SCP` tool outputs a pandas dataframe with the resids of polymers belonging to all aggregates in the system. Therefore, choosing other aggregates to analyse, such as the two smallest aggregates per time step (as illustrated in the tutorial), it is as easy as manipulating the pandas data frame to select the desired data.

Another pivotal function in the `make_micelle_whole` class is `make_cluster_whole`, which is its core function. This function performs the calculations to obtain the coordinates of the connected bond structure. The output of this function is a NumPy array with the atom coordinates. An example of how to run this function is illustrated in Figure S8. From here, it is clear that the only inputs required are: the resids on which the calculation will be performed, the time steps for the calculation and the resname of the molecules forming the structure. This function can accept multiple resnames as needed.

The last function of this class, `obtain_snapshot`, is designed to output an user-defined

```
from pysoftk.pol_analysis.tools.utils_mda import MDA_input
from pysoftk.pol_analysis.tools.utils_tools import *
from pysoftk.pol_analysis.clustering import SCP
from pysoftk.pol_analysis.make_micelle_whole import micelle_whole

#Select trajectory
topology='topology.tpr'
trajectory='trajectory.xtc'

#Select times to run micelle_whole on
start=0
stop=10001
step=1

#Load clustering resids
resids_total='results_clustering.parquet'

#Obtain the resids of the structure of interest
resids = micelle_whole(topology, trajectory).
    obtain_largest_micelle_resids(resids_total)

#Run make_cluster_whole function
atom_pos = micelle_whole(topology, trajectory).
    running_make_cluster_whole(['LIG'], resids, start, stop, step)
```

Figure S8: Code snippet showing how to use `running_make_cluster_whole`.

`pdb` file for the whole structure at the selected frame. This can be convenient if the user wants to input the structure into a different simulation box, for example, if the user wants to study the interaction of a micelle with a membrane, it can obtain the micelle pdb file with this function and then insert it into the membrane box. Figure S9 displays a code snippet showing how to use the function. The code in Figure S9 will write the pdb file into the directory and with the name specified in `results_name`.

```python
from pysoftk.pol_analysis.tools.utils_mda import MDA_input
from pysoftk.pol_analysis.tools.utils_tools import *
from pysoftk.pol_analysis.clustering import SCP
from pysoftk.pol_analysis.make_micelle_whole import micelle_whole

#Select trajectory
topology='topology.tpr'
trajectory='trajectory.xtc'

#Select times to run micelle_whole on
start=0
stop=10001
step=1

#Name of results file
results_name='Desktop/system_frame_2.pdb'

#Load clustering resids
resids_total='results_clustering.parquet'


#Obtain the resids of the structure of interest
resids = micelle_whole(topology, trajectory).
    obtain_largest_micelle_resids(resids_total)

#Run make_cluster_whole function
atom_pos = micelle_whole(topology, trajectory).
    running_make_cluster_whole(['LIG'], resids, start, stop, step)

#Obtaining pdb of the system at frame 2
snapshot = micelle_whole(topology, trajectory).obtain_snapshot(
    results_name, atom_pos[2], resids[2], ['LIG'], 2)
```

Figure S9: Code snippet showing how to use `obtain_snapshot`.

`rgyr`: radius of gyration

An example is provided in the Jupyter notebook tutorial `rgyr_mdanalysis_vs_pysoftk`, To illustrate the effectiveness of PySoftK in computing the radius of gyration with minimal user input, we compare the radius of gyration of a micelle broken across the PBC using MDAnalysis and PySoftK. The code snippet provided in Figure S10 demonstrates the ease with which the radius of gyration can be calculated for a micelle with varying numbers of polymers over the course of the simulation.

This function allows the user to easily calculate the radius of gyration of a structure that is not always formed by the same molecules throughout the simulation. It utilises the MDAnalysis function radius_of_gyration (pbc=True), but allows users to specify the atom positions and their corresponding resids on which to perform this calculation at each time step. Figure 3 in the main text shows the comparison between using the MDAnalysis radius of gyration function alone compared to the PySoftK rgyr, which captures the right radius of gyration of the micelle when computed on the whole coordinates from make_micelle_whole.

The input variables for the `rgyr` tool in Figure S10 are: the topology and trajectory of the simulation (`topology` and `trajectory`), the residue IDs of the molecules of interest (`resids`), the atomic positions (`atom_pos`), and the frames to analyze (`start`, `stop`, and `step`). The `resids` variable is a NumPy array containing the residue IDs of the molecules that belong to the structure of interest at each time step. In this way, it is very easy for the user to account for the varying conformation of the micelle. Also, `atom_pos` is the NumPy array containing the whole atom positions. It is worth noting, that `atom_pos` must include the coordinates of all the atoms contained in `resids`. The output `rgyr_micelle_whole` from Figure S10 is a NumPy array with the radius of gyration of the micelle over the selected time frames. The jupyter notebook tutorial named `rgyr_tutorial` provides a step-by-step tutorial for the radius of gyration calculation using PySoftK's `rgyr` tool.

```python
from pysoftk.pol_analysis.tools.utils_mda import MDA_input
from pysoftk.pol_analysis.tools.utils_tools import *
from pysoftk.pol_analysis.make_micelle_whole import micelle_whole
from pysoftk.pol_analysis.rgyr_micelle import rgyr

#Select trajectory
topology='topology.tpr'
trajectory='trajectory.xtc'

#Load clustering resids
resids_total='results_clustering.parquet'

#Select times to run micelle_whole on
start=0
stop=10001
step=1

#Run micelle_whole
resids = micelle_whole(topology, trajectory).
    obtain_largest_micelle_resids(resids_total)
atom_pos = micelle_whole(topology, trajectory).
    running_make_cluster_whole(['LIG'], resids, start, stop, step)

#Run rgyr calculation
rgyr_micelle_whole = rgyr(topology, trajectory).running_rgyr(
    resids, atom_pos, start, stop, step)
```

Figure S10: Code snippet showing how to use `rgyr`.

`ecc`: Eccentricity calculation

The input parameters of `ecc` are the same as `rgyr`, and the output is a NumPy array that contains the eccentricity values of the desired structure at each time step. The tutorial `ecc_tutorial` shows how to use this function.

Eccentricity, a metric quantifying a structure's deviation from a perfect sphere, serves as a useful tool for assessing the shape of spherical-like soft matter aggregates. The ecc tool calculates the eccentricity for any molecular structure by leveraging the MDAnalysis function moment_of_inertia() and employing the following formula:

$$\epsilon = 1 - \frac{I_{min}}{I_{mean}} \tag{1}$$

where ϵ is the eccentricity value, I_{min} is the minimum moment of inertia across all axis of the molecule(s), and I_{mean} is the mean moment of inertia over all axis of the molecule(s). A perfect sphere corresponds to $\epsilon = 0$, while increasing values indicate more oblong structures. Similar to the rgyr calculation, the ecc tool can account for varying number of molecules within the structure and by using the coordinates of the micelle having been corrected by make_micelle_whole as input, it ensures accurate calculations without artefacts from PBC. Figure S11 illustrates how ecc accurately computes the eccentricity of a micelle over time compared to the MDAnalysis strategy, since it uses the correct coordinate reconstruction across the PBC.

`spherical_density`

PySoftK's `spherical_density` tool empowers users to calculate spherical density over time, even for structures with fluctuating molecule numbers throughout the simulation. It efficiently computes the average density (over time) with respect to the distance from the center of mass of the molecular structure. This is carried out by the function `spherical_density`

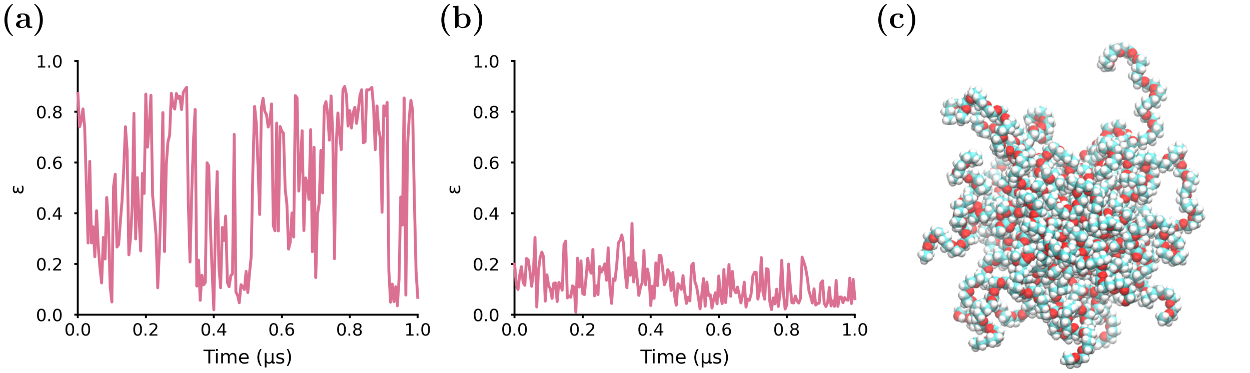

Figure S11: **Eccentricity calculation of polymer micelle.** Calculation of the eccentricity of a micelle solving Equation 1 over time with (a) only MDAnalysis moment_of_inertia() and (b) PySoftK ecc function. It is clear that ecc can easily use the correct polymers belonging to the cluster at each time step and the correct atom positions across the PBC to compute the eccentricity values, while the MDAnalysis function on its own, even with pbc=True is not able to compute them properly. (c) Snapshot of the micelle on which the eccentricity is being calculated, clearly it is slightly spherical, so the values from (b) are the correct ones. The micelle representation is not to scale. Polymer trajectories of diblock PEO-PMA polymer from *López-Ríos et al.*[5]

`_calculation` defined as:

$$\rho_{bin} = \frac{N_{particles}}{\frac{4}{3}\pi(R^3 - r^3)} \tag{2}$$

Where ρ_{bin} is the density per bin, $N_{particles}$ is the number of particles in that bin, R is the outer bin radius and r the inner bin radius. The user can define the number of bins and size of the bins to divide the space. It is recommended to pick a range of bins large enough to account for the whole extension of the molecular structure. It is important to keep in mind that optimizing the number of bins and their width is an iterative process. This function is very intuitive, as shown in Figure S12 where an example is provided.

Figure S12 shows that the inputs needed to run `spherical_density` are: the trajectory, the topology, the type of string selection for all the molecules of the molecular ensemble, the selection of all the molecules of the ensemble, the whole atom coordinates of all the molecules of the ensemble and the names of the component of the density calculation. For the string selection type, in this example the option `'resids '` is selected. However, note

```python
from pysoftk.pol_analysis.tools.utils_mda import MDA_input
from pysoftk.pol_analysis.tools.utils_tools import *
from pysoftk.pol_analysis.make_micelle_whole import micelle_whole
from pysoftk.pol_analysis.spherical_density import
    spherical_density

#Select trajectory
topology='topology.tpr'
trajectory='trajectory.xtc'

#Load clustering resids
resids_total='results_clustering.parquet'

#Select times to run micelle_whole on
start=0
stop=10001
step=1

#Run micelle_whole
resids = micelle_whole(topology, trajectory).
    obtain_largest_micelle_resids(resids_total)
atom_pos = micelle_whole(topology, trajectory).
    running_make_cluster_whole(['LIG'], resids, start, stop, step)

#Selecting molecule atoms for density calculation
names_total =['C00A', 'C009', 'C008', 'C007', 'C006', 'C005', '
    C004', 'C003', 'C002', 'C001', 'C000', 'C00L', 'C00O', 'C00P',
    'C00Q', 'C00R', 'C00S', 'C00T']

#Run density calculation
spherical_density_whole, binned_space = spherical_density(topology
    , trajectory).run_density_calc('resid ', resids, atom_pos,
    names_total)
```

Figure S12: Code snippet showing how to run the spherical density function.

that all different keywords available in MDAnalysis (i.e. 'resname', 'name', among others) can be employed as an input for this parameter.

The `resids` parameter in Figure S12 represents the selection of all molecules belonging to the same molecular structure (per time step) on which the density calculation will be performed. Here, `resids` is a list containing the resids of all the molecules that belong to the micelle. If the string selection parameter had been a different one, such as the atom name, instead of a list of resids, this parameter would have been a list of names. `atom_pos` is a NumPy array with the whole atom positions of molecules listed in `resids`. Finally, `names_total` are the atom names of the component for the density calculation, which needs to be a list of `str`. The output `spherical_density_whole` is a NumPy array with the average density over time per bin. The bins are stored in the array `binned_space`.

Examples of both spherical density calculations, for the micelle components and water, are illustrated in the tutorial `spherical_density`. This tutorial shows how to calculate and plot the density in both cases.

Complementary, the tutorial titled `intrinsic_density_tutorial` covers how to run the intrinsic density of the hydrophobic component of a polymer micelle and the water of the system with PySoftK's intrinsic density tools.

Molecular-scale interactions analysis

`contacts`: Quantification of intermolecular interactions.

Figure S13 presents a code snippet illustrating the usage of the `contacts` tool. It requires several input files: the topology and trajectory of the simulation, the resids of the molecules on which the analysis will be performed on (`resids`), the whole atomic coordinates of the molecular ensemble (`atom_pos`), the atom names for both groups involved in the contact calculation (`MA_names`, `MA_names`) and the cutoff distance (`cutoff`). In Figure S13, intermolecular contacts are calculated between atoms of the same type, which is why `MA_names` is selected

```
from pysoftk.pol_analysis.tools.utils_mda import MDA_input
from pysoftk.pol_analysis.tools.utils_tools import *
from pysoftk.pol_analysis.make_micelle_whole import micelle_whole
from pysoftk.pol_analysis.contact_analysis import contacts

#Select trajectory
topology='topology.tpr'
trajectory='trajectory.xtc'

#Load clustering resids
resids_total='results_clustering.parquet'

#Select cutoff contact distance
cutoff=10

#Select times to run micelle_whole on
start=0
stop=10001
step=1

#Select atom contact names
MA_names = ['C027', 'C023', 'C021', 'C02H', 'C02L', 'C02P',
    'C02T', 'C02X', 'C00U', 'C00R', 'C00P', 'C00L']

#Run micelle_whole
resids = micelle_whole(topology, trajectory).
    obtain_largest_micelle_resids(resids_total)
atom_pos = micelle_whole(topology, trajectory).
    running_make_cluster_whole(['LIG'], resids, start, stop, step)

#Run contacts calculation
contacts_matrix = contacts(topology, trajectory).run_contacts_calc
    (resids, atom_pos, MA_names, MA_names, cutoff)
```

Figure S13: Code snippet showing how to run the `contacts` function.

for both contact groups. The output of the `contacts` class, in this case `contacts_matrix`, is a contact matrix containing the total number of intermolecular contacts between atoms of both contact groups. Rows correspond to atoms belonging to the first contact group, and columns correspond to the second contact group. It is important to note that to ensure consistency in performing this analysis, the frames from the user-provided trajectory employed in `SCP` and `make_micelle_whole` need to be the same, as depicted in Figure S13. For a step-by-step guide on using the `contacts` tool and recommendations for result plotting, refer to the tutorial named `contacts_tutorial`.

One thing to consider when using this tool is that the values of the distance cutoff used to characterise the interactions of different types of molecules will vary. The values for these cutoffs can be determined from radial distribution functions between the specific atoms of the two molecules used in the calculation or through an analysis of the minimum distance between these atoms on two molecules that are known to have aggregated during the simulation. Values of the distance cutoff have been found to be between 4 Å and 7 Å as shown elsewhere.[5,8,9] A visual representation of intermolecular contacts between two poly(ethylene oxide) (PEO) - poly(methyl acrylate) (PMA) polymers within a micelle is shown in Figure S14 (a). The contacts class can utilize the output of the make_micelle_whole tool as an input. This ensures that the distances between atoms within the molecules are calculated correctly when accounting for the periodic boundary effects within the system. Figure S14 (b) shows a heatmap representation of the normalized matrix generated by contacts, which shows the intermolecular EO-MA interactions of PEO-PMA polymers.

`RSA`: Ring Stacking Analysis

The code snippet in Figure S15 shows how to use this function. This code illustrates that the required input parameters are the topology and trajectory of the simulation, a ring-ring distance cutoff (`dist_cut`), a maximum angle between ring units (`angle_cut`), and

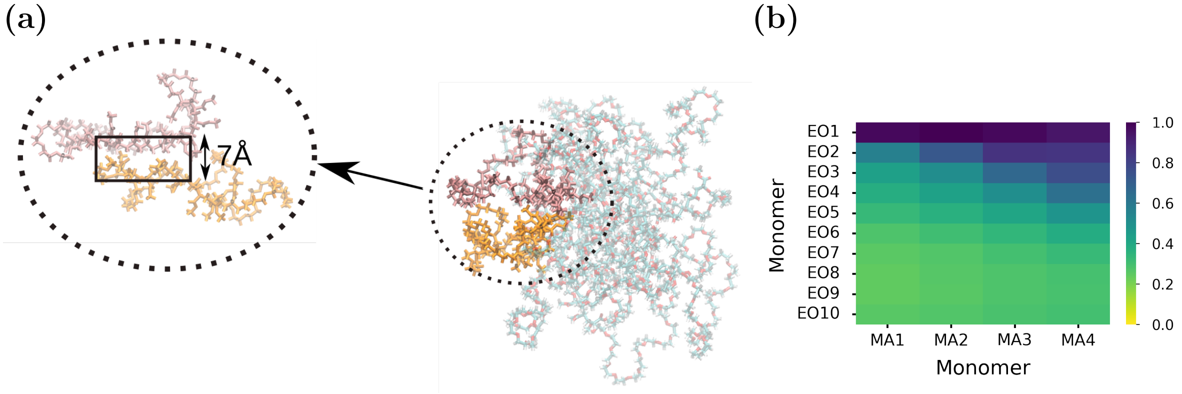

Figure S14: **Normalized contacts output.** (a) Snapshot of two cyclic polymer within a micelle that are in contact. The inter-molecular distance of atoms in the black rectangular box is 7 Å. These inter-molecular interactions would be picked up by the contacts algorithm as contacts, if the distance cutoff specified is 7 Å. (b) Output of the calculation of the intermolecular contacts of PEO-PMA polymers forming a micelle. The output matrix is represented as a heatmap. From here it is clear that the rows and columns represent the contact groups of the calculation. Polymer trajectories of cyclic PEO-PMA polymer from *López-Ríos et al.*[5]

the name of the output file name (`output`). The cutoff distance represents the maximum spatial separation allowed between aromatic units for the calculation of their stacking angle. Based on previous work analyzing the ring center-of-geometry pair distribution function in a conjugated polymer melt system,[10] we set a cutoff distance of 10 Å for identifying potential ring stacking events. This cutoff value corresponds to the observed plateau in the pair distribution function, which exhibited a peak at 6 Å and reached a plateau at 10 Å (Figure 2(b) of the mentioned reference). Similarly, the angle cutoff value will depend on the system and the properties of interest. The angle cutoff defines the angle range between two rings that is counted as stacking (angle between rings either smaller than the cutoff angle or larger than $\pi - cutoff$). The default recommended value is 20°,[10] but for very tight ring stacking, smaller values can be used. The output of the `RSA` tool is a pandas dataframe with all the resids of the polymers that are stacked and also the pdb files of pairs of stacked polymers. Furthermore, the `RSA` class has another function `find_several_rings_stacked` that uses as input the output from the rings stacking calculation and outputs the network of polymers that are interacting via ring stacking. It creates a graph object ($G(V, E)$) in the same manner

that was defined in `SCP` class to keep track of the polymers that are connected. The tutorial titled `RSA_tutorial` shows how to run both RSA functions.

```
from pysoftk.pol_analysis.tools.utils_mda import MDA_input
from pysoftk.pol_analysis.tools.utils_tools import *
from pysoftk.pol_analysis.ring_ring import RSA

#Select trajectory
topology='topology.tpr'
trajectory='trajectory.xtc'

#Select output name
results_name='results.parquet'

#Define angle and distance cutoff
dist_cut=5
angle_cut=30

#Select frames to run the clustering on
start=0
stop=10001
step=1

#Run clustering
rsa = RSA(topology, trajectory).stracking_analysis(dist_cut, angle_cut,
    start, stop, step, results_name)
```

Figure S15: Code snippet showing how to run the `RSA` tool.

solvation

The code snippet in Figure S17 shows how to use this function. Thus, as it can be seen in Figure S17 the required inputs for this function are: the topology and trajectory files (`water_topology, water_trajectory`), the frames for the calculation (`start, stop,`

(a) **(b)**

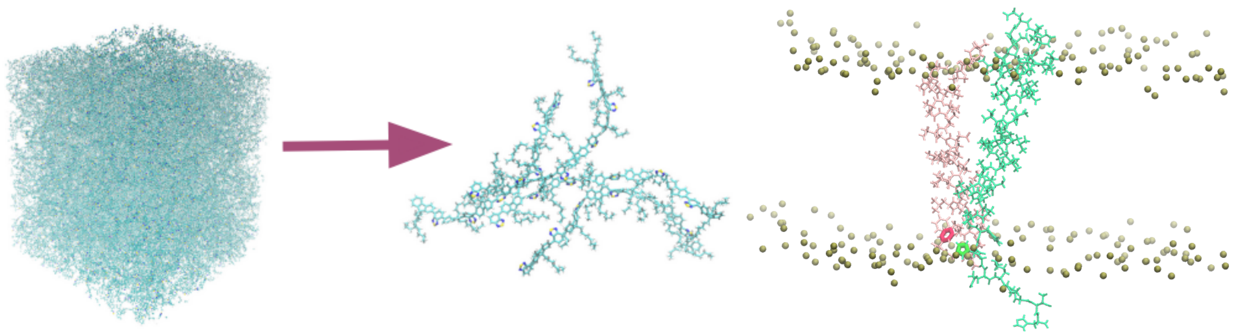

Figure S16: **Example of ring-ring stacking calculation using the RSA class on (a) a polymer melt and (b) on the TREM12 and DAP12 protein.** In (a) the RSA is able to obtain ring stacking in complicated and large system. The purple arrow points to a subset of polymers interacting in the amorphous phase via ring stacking. This cluster has been identified with the RSA tool. (b) RSA applied to determine ring stacking events that drive protein-protein interactions. TREM12 is shown in pink and DAP12 in green. An observed ring stacking interaction are denoted by the bold representation. Phosphate groups of the membrane are colored in dark green. Representations are not to scale. Trajectory in (a) is from *Ziolek et al.*[10] and (b) from *Zhong et al.*[?]

`step`), the resids of the molecules for the solvation calculation (`resids`), the whole atomic position of all molecules in `resids` (`atom_pos`), the name of the solvent molecules (`water_name`), the selected polymer atoms for the solvent calculation (`molecule_names`) and the cutoff distance to compute the solvation shell (`cut`). The output, in this case `solvation_number`, is a list where each entry represents the solvation of the selected atoms across all molecules in the system. There are as many entries as the frames in the calculation. The tutorial named `solvation_tutorial` shows step by step how to use the `solvation` function to calculate the solvation of specific atoms of polymers belonging to the same micelle, and how to further process and plot the output data.

Currently, there is no readily available open-source software that can be used in any soft-matter system. MDAnalysis has the class MDAnalysis.analysis.waterdynamics, but it focuses on the dynamics of water and the interactions of water with other molecules via hydrogen bonds. Figure S18 shows the average solvation number calculated for all hydrophobic MA monomers of a PEO-PMA polymeric micelle.

```python
from pysoftk.pol_analysis.tools.utils_mda import MDA_input
from pysoftk.pol_analysis.tools.utils_tools import *
from pysoftk.pol_analysis.make_micelle_whole import micelle_whole
from pysoftk.pol_analysis.solvation import solvation

#Select trajectory
topology='topology.tpr'
trajectory='trajectory.xtc'
resids_total='results_clustering.parquet'#Select times to run micelle_whole on
start=0
stop=10001
step=1
#Run micelle_whole
resids = micelle_whole(topology, trajectory).obtain_largest_micelle_resids(resids_total)
atom_pos = micelle_whole(topology, trajectory).running_make_cluster_whole(['LIG'], resids, start, stop, step)
#Selecting molecule atoms to study solvation
molecule_names=['000A', '000D', '000C', '001G', '001F']
#Oxygen solvent name
water_name=['OW']
#Cutoff
cut=4.5
#Run solvation
solvation_number=solvation(topology, trajectory).solvation_calc_run(start, stop, step, resids, atom_pos, water_name, molecule_names, cut)
```

Figure S17: Code snippet showing how to run the `solvation` class.

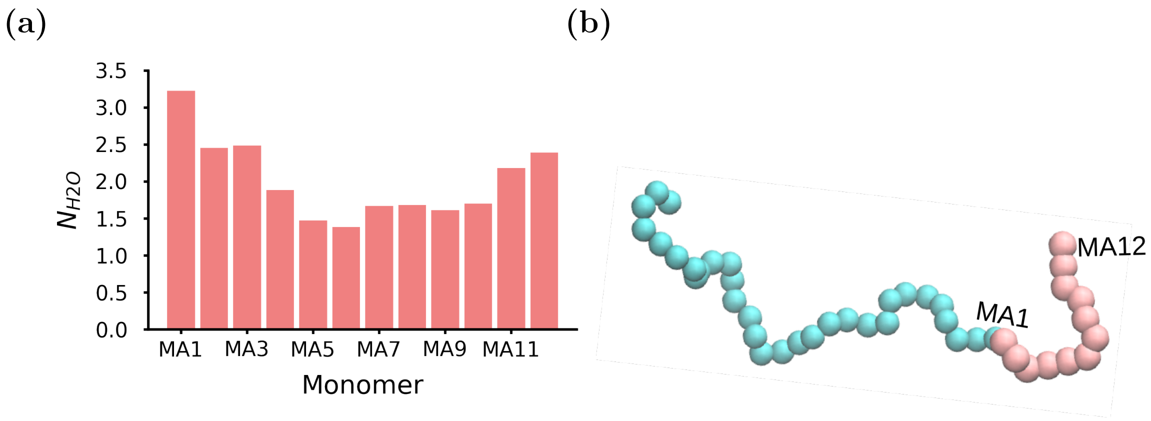

Figure S18: **Solvation calculation of polymer micelle.** Average solvation of a diblock BCP micelle. (a) Average over time of water coordination numbers for all monomers of hydrophobic block. (b) Snapshot of the diblock polymer being studied. PMA in pink and PEO in blue. Polymer representation is not to scale. Polymer trajectories of PEO-PMA polymer from *López-Ríos et al.*[5]

The solvation class operates similarly to the contacts class, where distances between selected atoms of the molecules and specific solvent atoms are computed. If the distance is shorter than a user-defined cutoff, the selected atom in a molecule of interest is considered to be solvated. The inputted cut-off distance can be used to represent a given solvation shell of the molecule (e.g. first or second solvation shell) depending on what is of interest to the user. When using water as the solvent, it is recommended to select just the oxygen water atoms to speed up calculations. Consistent with the other tools in PySoftK v1.0, the solvation code seamlessly handles the varying number of molecules and the correct atomic coordinates at each time step.

`tools`

All PySoftK analysis classes use the `pysoftk.pol_analysis.tools` module, which has two classes, `MDA_input` and `utils_tools`. The `MDA_input` class is used to load the trajectory as an MDA universe for all calculations. On the other hand, `utils_tools` contains all recurrent functions that the analysis classes use in their computations. Therefore, both `tools.utils_mda` and `tools.utils_tools` need to be imported whenever a PySoftK analysis

tool is used.

References

(1) Bougueroua, S.; Spezia, R.; Pezzotti, S.; Vial, S.; Quessette, F.; Barth, D.; Gaigeot, M.-P. Graph theory for automatic structural recognition in molecular dynamics simulations. *The Journal of chemical physics* **2018**, *149*.

(2) Ziolek, R. M.; Smith, P.; Pink, D. L.; Dreiss, C. A.; Lorenz, C. D. Unsupervised learning unravels the structure of four-arm and linear block copolymer micelles. *Macromolecules* **2021**, *54*, 3755–3768.

(3) Rhys, N. H.; Al-Badri, M. A.; Ziolek, R. M.; Gillams, R. J.; Collins, L. E.; Lawrence, M. J.; Lorenz, C. D.; McLain, S. E. On the solvation of the phosphocholine headgroup in an aqueous propylene glycol solution. *The Journal of chemical physics* **2018**, *148*.

(4) Hagberg, A.; Conway, D. Networkx: Network analysis with python. *URL: https://networkx. github. io* **2020**,

(5) De Castro, R. L.-R.; Ziolek, R.; Lorenz, C. Topology-Controlled Self-Assembly of Amphiphilic Block Copolymers. **2023**,

(6) Michaud-Agrawal, N.; Denning, E. J.; Woolf, T. B.; Beckstein, O. MDAnalysis: a toolkit for the analysis of molecular dynamics simulations. *Journal of computational chemistry* **2011**, *32*, 2319–2327.

(7) Santana-Bonilla, A.; Lopez-Rios de Castro, R.; Sun, P.; Ziolek, R. M.; Lorenz, C. D. Modular Software for Generating and Modeling Diverse Polymer Databases. *Journal of Chemical Information and Modeling* **2023**,

(8) Ulmschneider, M. B.; Doux, J. P.; Killian, J. A.; Smith, J. C.; Ulmschneider, J. P. Mechanism and kinetics of peptide partitioning into membranes from all-atom simulations of thermostable peptides. *Journal of the American Chemical Society* **2010**, *132*, 3452–3460.

(9) Sun, X.; Feng, Z.; Hou, T.; Li, Y. Mechanism of graphene oxide as an enzyme inhibitor from molecular dynamics simulations. *ACS applied materials & interfaces* **2014**, *6*, 7153–7163.

(10) Ziolek, R. M.; Santana-Bonilla, A.; Lopez-Rios de Castro, R.; Kuhn, R.; Green, M.; Lorenz, C. D. Conformational heterogeneity and interchain percolation revealed in an amorphous conjugated polymer. *ACS nano* **2022**, *16*, 14432–14442.
